# Effect of sub-MIC values of metronidazole, ciprofloxacin, and imipenem on the growth and toxin production in *Clostridioides difficile*

**Published:** 2019

**Authors:** Farahnaz Sadat Shayegan Mehr, Masoumeh Azimirad, Seyedeh Nazanin Mansouri Gilani, Ayub Ghafurian, Abbas Yadegar

**Affiliations:** 1 *Foodborne and Waterborne Diseases Research Center, Research Institute for Gastroenterology and Liver Diseases, Shahid Beheshti University of Medical Sciences, Tehran, Iran*; 2 *Gastroenterology and Liver Diseases Research Center, Research Institute for Gastroenterology and Liver Diseases, Shahid Beheshti University of Medical Sciences, Tehran, Iran*; 3 *Basic and Molecular Epidemiology of Gastrointestinal Disorders Research Center, Research Institute for Gastroenterology and Liver Diseases, Shahid Beheshti University of Medical Sciences, Tehran, Iran *

**Keywords:** Clostridioides difficile, Toxin production, Bacterial growth, Antibiotics, sub-MIC

## Abstract

**Aim:**

This study intends to investigate the effect of sub-minimum inhibitory concentration (sub-MIC) of metronidazole, ciprofloxacin, and imipenem on the growth and toxin production in *Clostridioides difficile*.

**Background:**

*C. difficile* is the most common causative agent of hospital-acquired diarrhea. Toxin production in *C. difficile* appears to be a critical process for induction of the disease. Several factors such as antibiotics can facilitate growth and toxin production in *C. difficile*.

**Methods:**

Five *C. difficile* strains were grown with and without sub-MIC concentrations of metronidazole, ciprofloxacin, and imipenem (0.5x MIC). The bacterial growth was measured by density at OD620 nm in 0, 4, 8, 12 and 24 h post inoculation. Toxin production was detected using ELISA in culture supernatants as well as in cell pellet.

**Results:**

The five strains showed minor growth variations in the presence and absence of antibiotic sub-MIC values, except for metronidazole, in which the sub-MIC concentration reduced the growth rate of the resistant isolate in comparison with the control without antibiotic. There were no significant variations in the levels of toxin production with the sub-MIC values of antibiotics examined in comparison with antibiotic-free controls. However, the amount of toxin production in the culture supernatant was greater than in the cell pellet.

**Conclusion:**

The findings of this study suggested that sub-MIC concentrations of antibiotics may have minor effects on bacterial growth and toxin production of *C. difficile*. Taken together, these findings suggest that presence of antimicrobial agents increased expression levels of certain genes, particularly virulence genes, which may help *C. difficile* to survive.

## Introduction

*Clostridioides* *difficile *(formerly *Clostridium difficile*) is a Gram-positive, anaerobic, spore-forming bacterium, which has emerged as an increasingly important nosocomial pathogen and the prime causative agent of antibiotic-associated diarrhea (AAD) and pseudomembranous colitis (PMC) in humans (1, 2). The intestinal colonization of *C. difficile* is essentially triggered following the elimination of beneficial resident microbiota through long-term use of certain antibiotics (3). The colonization process involves a variety of cell surface-associated proteins. Overgrowth of hypervirulent strains of *C. difficile* in the gut can lead to production of toxin A and toxin B as their major virulence factors. The pathological manifestations related to these toxins are disruption of epithelial barrier integrity, fluid loss, intestinal inflammation, and tissue destruction (4). *C. difficile* toxins (A and B) are among the largest bacterial toxins known with potent cytotoxicity and enterotoxicity (4). Exposure to the antibiotics is the most important risk factor for development of *C. difficile* infection (CDI) (5, 6). Nearly all antibiotics are thought to be able to trigger CDI, but broad-spectrum agents such as cephalosporins, clindamycin, and fluoroquinolones are the agents often reported to be associated with *C. difficile*-associated disease (CDAD) (7). Several factors, such as temperature, biotin limitation, amino acid concentration, glucose concentration, and alteration of the oxidation-reduction potential can affect toxin production in *C. difficile* (8-10). So far, a few studies have been performed on the physiological effects of sub-MIC concentrations of antibiotics in gene expression and toxin production of *C. difficile* (11-13). Thus, this study aimed to evaluate the effects of sub-MIC concentrations of three antibiotics including metronidazole, ciprofloxacin, and imipenem on the growth and production levels of toxins A and B of *C. difficile* clinical isolates. 

**Table 1:** The MIC values for the antibiotics tested against 5 clinical isolates of *C. difficile *involved in this study

Antibiotic agent	Isolate 1(µg/ml)	Isolate 2(µg/ml)	Isolate 3(µg/ml)	Isolate 4(µg/ml)	Isolate 5(µg/ml)
Metronidazole	32	16	16	16	16
Ciprofloxacin	8	32	8	32	32
Imipenem	16	16	8	8	32

**Figure 1:**
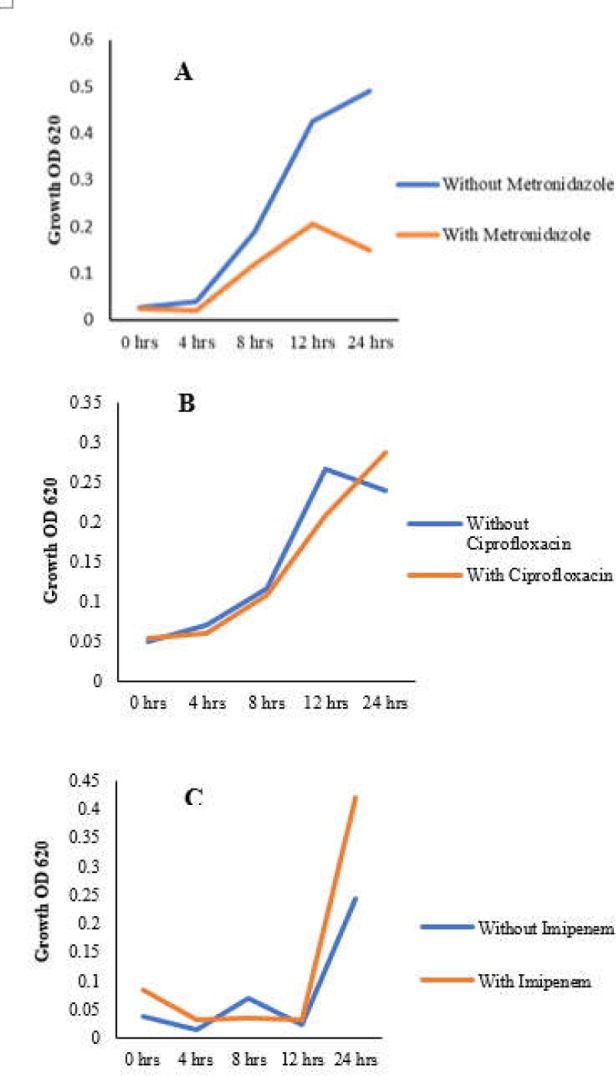
Mean growth of the studied isolates in the media containing antibiotic in comparison with antibiotic-free controls. Growth of the five isolates were examined at OD_620_ nm in time periods of 0, 4, 8, 12 and 24 hours when exposed to 1/2 MICs of the metronidazole (A), ciprofloxacin (B), and imipenem (C). Minor growth variations were observed in the presence and absence of 1/2 MICs except for metronidazole, in which the sub-MIC concentration reduced the growth rate of the resistant isolate in comparison with the antibiotic-free control

## Methods

***C. difficile*** **isolates**

Five clinical isolates of toxigenic *C. difficile* (TcdA^+^ and TcdB^+^), which had been preserved in the Anaerobic Laboratory at Research Institute for Gastroenterology and Liver Diseases, were used in this study (14). All isolates were stored in cooked meat broth (Himedia, India) at 4°C. Antibiotic susceptibility of the strains was examined against three antibiotics including metronidazole (Serva, USA), ciprofloxacin (Himedia, India), and imipenem (Glaxo, United Kingdom) using the agar dilution method according to the CLSI guidelines (15). Breakpoint values of ≥32 μg/ml for metronidazole, ≥8 μg/ml for ciprofloxacin, and ≥16 μg/ml for imipenem were considered as definitive criteria for the interpretation of resistant isolates.

**Growth curves**

The isolates were sub-cultured from cooked meat broth onto *C. difficile *medium (Mast, London, UK) supplemented with 7% horse blood and* C. difficile *selective supplement (Mast, UK) consisting of D-cycloserine (250 mg/ liter), cefoxitin (8 mg/liter), and lysozyme (5 mg/liter). The cultured plates were incubated at 37°C for at least 48-72 h under anaerobic conditions (85% N_2_; 10% CO_2_ and 5% H_2_) generated by Anoxomat® Gas Exchange System (Mart Microbiology BV, Holland). After incubation and a purity check on *C. difficile *medium, one colony was inoculated in 5 ml pre-reduced thioglycolate broth (Pronadisa, Spain) for 24 h. The bacterial density was captured by McFarland standards (McFarland 1=3 x 10^8^ cfu/ml). By measuring OD_550_ nm, an inoculum of 3 x 10^6^ cells/ml was incubated in 150 ml thioglycolate broth containing the appropriate antibiotic concentration (1/2 MIC). An antibiotic-free control was used for each strain. Bacterial growth was measured by reading bacterial density at OD_620_ nm in 0, 4, 8, 12 and 24 h post inoculation. For each time point, samples were taken as follows: (i) 1 ml for measuring bacterial growth in OD_620_ nm; (ii) 1 ml for evaluation of A and B toxins by ELISA method. Samples (ii) were centrifuged for 5 min at 6000 g and the supernatants were filtered (0.22 mm) and transferred to new 1.5 ml tubes. The remaining pellets were stored separately. Both supernatants and pellets were stored at -70 °C until toxin analysis.

**Toxins A and B ELISA**

Samples collected from cultures of *C. difficile *in thioglycolate broth were used to quantify the production of both toxins A and B by ELISA. To perform this test, samples at different time points were measured by ELISA using the collected supernatants and pellets via the *Clostridium difficile* Antigen kit (Generic Assays, Germany) according to the manufacturer’s instructions. Prior to this test, 500 µl of freshly prepared lysozyme was added to the pellet and incubated for 30 min at 37°C. One hundred microliters of each sample were used in ELISA at a wavelength of 420 nm. 

## Results

**Growth of** ***C. difficile*** **isolates in the presence and absence of antibiotics**

The MIC values for the antibiotics tested against 5 *C. difficile* clinical isolates are shown in Table 1. Accordingly, the growth rate of *C. difficile* isolates was measured in the presence and absence of sub-MIC values of metronidazole (16 μg/ml), ciprofloxacin (4 μg/ml), and imipenem (16 μg/ml) at different time points of 0, 4, 8, 12, and 24 hours post inoculation. 

As displayed in Figure 1, minor growth variations were observed in the presence and absence of antibiotic sub-MIC values during various time points, except for metronidazole, in which the sub-MIC concentration reduced the growth rate of the resistant isolate in comparison with the antibiotic-free control.

**The effect of antibiotics on the expression level of** ***C. difficile*** **toxins**

**Figure 2:**
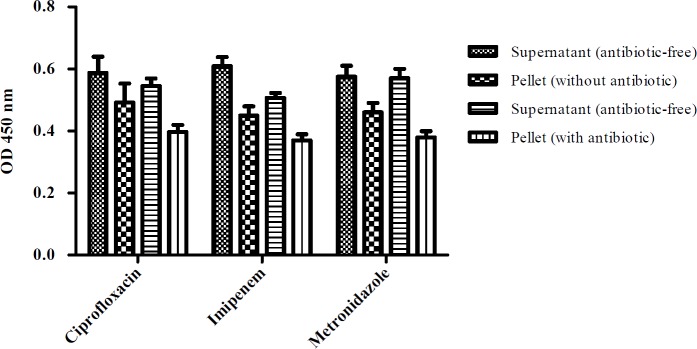
Mean toxin production in the supernatant and cell pellet of the studied isolates after 24 hours in the media containing antibiotics in comparison with antibiotic-free controls

The levels of toxins A and B production of *C. difficile* isolates in the culture supernatant and pellet when exposed to the antibiotics were also measured by ELISA after 24 hours of inoculation. As shown in Figure 2, there was no significant changes in the levels of toxin production in the presence of each antibiotic tested in comparison with antibiotic-free controls. However, as excepted the amount of toxin production in the culture supernatant was greater than in the cell pellet.

**Statistical analyses**

The level of toxin production and growth rate of *C. difficile* isolates in the presence or absence of antibiotic were analyzed via GraphPad Prism software (GraphPad Software Inc., La Jolla, CA, USA). Differences were considered statistically significant at P values < 0.05.

## Discussion

Over the last decade, the number of hypervirulent and antibiotic resistant *C. difficile* strains has increased and distributed worldwide. Furthermore, the emergence of recurrent CDI (rCDI) remains a significant challenge in the disease management and is a major cause of CDI-related mortality (16).

It has been estimated that approximately 22% and 14% of the CDI eradication failures as well as 27% and 24% of rCDI, are associated to treatment with metronidazole and vancomycin, respectively (17). The depletion of the normal protective gut microbiota by broad-spectrum antibiotics is a prerequisite for CDI. It has been proposed that use of broad-spectrum antibiotics can facilitate the development of CDI through disruption of further protective gut microbiota in humans. Moreover, long-term consumption of antibiotics leads to suppression of colonization resistance provided by beneficial microbiota, and promote the overgrowth and expansion of harmful bacteria (11, 18). Therefore, the main objective of the current study was to evaluate the impact of sub-MIC concentrations of various antibiotic on the growth rate and toxin production of *C. difficile* clinical isolates.

Our results revealed that sub-MIC values of ciprofloxacin and imipenem had small effects on the growth rate of resistant *C. difficile* isolates during the indicated time points in this study. However, the sub-MIC concentration of metronidazole lowered the growth rate of the resistant isolate in comparison with the antibiotic-free control. In a similar study by Drummond et al., *C. difficile *strains were cultured in sublethal concentrations of antibiotics (1/2, 1/4 and 1/8 MIC) including metronidazole, vancomycin, amoxicillin, clindamycin, cefoxitin, and ceftriaxone over 104 hours, with growth and production of toxin A measured (11). They observed that effects of sub-MIC levels of antibiotics on the kinetics of bacterial growth varied with both the antibiotic and strain. In addition, subinhibitory concentrations of antibiotics tended to delay the growth rate of the bacteria by increasing the lag period, especially at the highest concentration of antibiotic (1/2 MIC), except for strain NCTC 11223 in the presence of clindamycin. With regard to clindamycin, when the sub-MIC levels of this antibiotic were added, *C. difficile* growth was not affected. Our findings are in agreement with these data, where there was little variation in the bacterial growth between the studied isolates. An explanation for this may be the successful adaptation of *C. difficile* strains to certain antibiotic agents which let them function and grow as normal. Further, some of the *C. difficile* strains may have the resistance determinants, which make them resistant to certain antibiotics. On the other hand, *C. difficile* strain 630 carries the *ermB* gene (encoding an RNA methyltransferase) which is responsible for resistance to macrolide, lincosamide, and streptogramin B (MLS), but its growth is affected by the sub-MIC concentrations of clindamycin (19). However, the reasons for this heterogeneity are not well understood.

There is controversial data on the impact of subinhibitory concentrations of antibiotics on toxin production of *C. difficile* strains (11, 20, 21). A few studies have demonstrated that antibiotics can affect the expression of *C. difficile* toxin and facilitate its production (22, 23). Based on our results, no significant variations were observed in the levels of toxin production with the sub-MIC values of antibiotics examined in comparison with antibiotic-free controls. In contrast, Gerber et al. examined the toxin production levels of four *C. difficile* strains when grown with and without sub-MIC concentrations of metronidazole, vancomycin, clindamycin, and linezolid (0.5x MIC) (21). They found that the four strains showed similar growth, but different levels of toxin production in the absence of antibiotics. They also indicated that toxin production occurred at a late stage of bacterial growth in antibiotic-free control, while antibiotic-exposed strains showed earlier toxin production. Additionally, all of the antibiotics tested except clindamycin increased the transcription rate of toxin A and B genes. Furthermore, Honda et al. observed that certain antibiotics increased the production of the two *C. difficile* toxins. They showed that clindamycin concentration of 1 µg/ml (one-tenth of the MIC) and cephaloridine (1-25 µg/ml, one eighth of the MIC) stimulated the production of two *C. difficile* toxins by 16 and 8-fold, respectively (24). Further, it seems that some antibiotics at low concentrations have the ability to influence the transcriptional modulation of bacterial gene expression and toxin genes (25, 26).

In conclusion, our findings indicated that sub-MIC values of antibiotics may have little effects on bacterial growth and toxin production of *C. difficile*. Taken together, these findings suggest that, under harsh conditions such as the presence of antimicrobial agents, increased expression levels of certain genes, particularly virulence genes, may help *C. difficile* to survive. However, it is difficult to interpret the observations and effects of antibiotics at sub-MIC values *in vitro* experiments with reference to their clinical use under *in vivo* conditions. Thus, further studies using a larger number of *C. difficile* isolates and extended sub-MIC values of various antibiotics can help better translate the experimental results to *in vivo* conditions, and provide practical advice on clinical therapy of CDI.
